# Beyond Hirschsprung: outcomes of symptomatic infants after exclusion of Hirschsprung disease on rectal suction biopsy

**DOI:** 10.1007/s00383-025-06219-z

**Published:** 2025-10-23

**Authors:** Sarah Metzger, Isabella Robbiani, Sasha J. Tharakan, Noëmi Zweifel, Carsten Posovszky, Ueli Moehrlen, Hannah R. Neeser

**Affiliations:** 1 Department of Pediatric Surgery, University Children’s Hospital Zurich, Zurich, Switzerland; 2 Department of Pediatric Gastroenterology, University Children’s Hospital Zurich, Zurich, Switzerland

**Keywords:** Hirschsprung disease, Defecation disorder, Laxatives, Infancy, Rectal suction biopsy

## Abstract

**Purpose:**

Rectal suction biopsies (RSB) to exclude Hirschsprung disease (HD) are performed on a low threshold in infants with defecation problems. This leads to a significant number of symptomatic infants with excluded HD. We intend to characterize them and provide a perspective on their clinical outcome.

**Methods:**

A retrospective chart review was performed in 90 infants who underwent RSB at our institution from 2011 to 2022. In 54 infants, HD was excluded. Thirty one of those infants met the inclusion criteria and were further analyzed.

**Results:**

Treatment was initiated prior to biopsies with rectal irrigations in 71% (*n* = 22), suppositories in 3% (*n* = 1), oral laxatives in 23% (*n* = 7), and dietary modifications in 3% (*n* = 1). At last follow-up, 81% (*n* = 25) of patients had no further need for treatment, and 19% still regularly used oral laxatives or suppositories (*n* = 6). In three patients, another gastroenterological diagnosis was found. Four patients were re-referred to the gastroenterological clinic later in childhood.

**Conclusion:**

Defecation problems in early infancy will resolve in most patients with excluded HD without any further treatment. However, some patients will continue to have gastrointestinal symptoms and may need to take laxatives long-term.

## Introduction

Defecation problems in infants are common, affecting 12–15% or more, and lead many parents to consult their pediatrician for symptoms associated with their baby's bowel habits [1, 2]. In many cases, no organic cause for the defecation difficulties is identified, thus meeting the diagnostic criteria for a functional gastrointestinal disorder, such as functional infant dyschezia or functional constipation [2, 3]. However, some infants may have an underlying organic cause for their defecation difficulties, such as dietary protein intolerance, celiac disease, hypothyroidism, cystic fibrosis, anal achalasia or motility disorders, such as Hirschsprung disease (HD) [3].

HD is one of the most common congenital disorders of intestinal motility [4, 5] and early diagnosis is crucial to avoid serious complications [6]. The diagnosis of HD is made by demonstrating the absence of ganglion cells in the distal rectum on histopathological examination [7]. Symptoms suggestive for HD are among others a delayed passage of meconium, abdominal distension, and bilious emesis [8, 9]. The gold standard for diagnosing HD in infants is rectal suction biopsies (RSB) [3, 10] and the complication rates for RSB are low, being less than 1% even in preterm infants [7, 11]. Consequently, the threshold for obtaining biopsies in symptomatic infants is low with a considerable number of negative HD biopsies [7, 8, 12, 13]. However, little is known regarding the outcome of symptomatic infants with histologically excluded HD. Due to this current gap in knowledge, pediatricians and pediatric surgeons face difficulties to counsel families on the anticipated outcome of their child’s symptoms after the exclusion of HD.

In this retrospective study, we therefore aim to characterize symptomatic infants with excluded HD and to provide their families with an outlook on the clinical outcome.

## Materials and methods

### Data collection

This is a retrospective single-center cohort study including all patients who underwent RSB for suspected HD. The case group comprised infants with relevant symptoms of a defecation disorder who underwent RSB for suspected HD and in whom the diagnosis of HD was excluded based on the presence of ganglion cells in histopathological examination compared to infants with confirmed HD. Therefore, we performed a retrospective chart review of all patients who had at least one RSB performed at our institution between January 2011 and May 2022 and who have documented consent. We excluded all patients with inconclusive biopsy results, without clinical follow-up or with a known underlying condition which could possibly explain the clinical presentation.

The study was approved by the local ethics committee (BASEC 2022-01007).

The following data were retrieved from the patients’ medical records: demographic variables, clinical presentation, diagnostic work-up with particular attention to the histopathological results of the rectal suction biopsies, treatment, and clinical outcome. Symptoms at initial presentation were assessed as present/reported and not present/not reported, given the high probability that if a symptom is not mentioned in the patient chart, it can be assumed to have not been present. Follow-up time was defined as time from first biopsies to last contact with either our pediatric surgery or gastroenterology department.

At our institution, the majority of surgeons initiate treatment for potential HD in patients prior to RSB, based on the presenting symptoms. If there is a high degree of suspicion for HD, rectal irrigations are initiated. Irrigations are administered twice daily with a sodium chloride solution, followed by the insertion of an age-appropriate anal dilator to facilitate evacuation of the irrigation fluid. However, some surgeons, particularly in the case of slightly older infants in good general health, opt to continue treatment with suppositories or laxatives until a diagnosis is made or ruled out. Once HD has been ruled out, rectal irrigations are tapered. All other treatments are adapted based on the clinical course and patient’s needs by the treating surgeon or pediatric gastroenterologist. Once an organic cause is excluded with high likelihood and symptoms improve management is usually transferred back to the general practitioner.

Treatment strategies were classified into the following categories: rectal irrigations, suppositories, oral laxatives, dietary modifications, and no treatment. If two different treatments were administered concurrently, only the more invasive treatment was accounted for.

### Statistical analysis

Statistical analysis was performed with the R statistical software (version 4.3.0) [14] and R studio (version 2024.04.2 + 764) [15]. The following additional packages were used: openxlsx, readxl [16, 17]. Correlations between variables were calculated using Fisher’s exact test for discrete data and Mann–Whitney *U* test for continuous data. We considered the results to be statistically significant when *p* was < 0.05.

Microsoft Excel and Word (Microsoft Corporation, Redmond, WA, USA) were used to generate figures and graphs.

## Results

### Patient characteristics

**Fig. 1:**
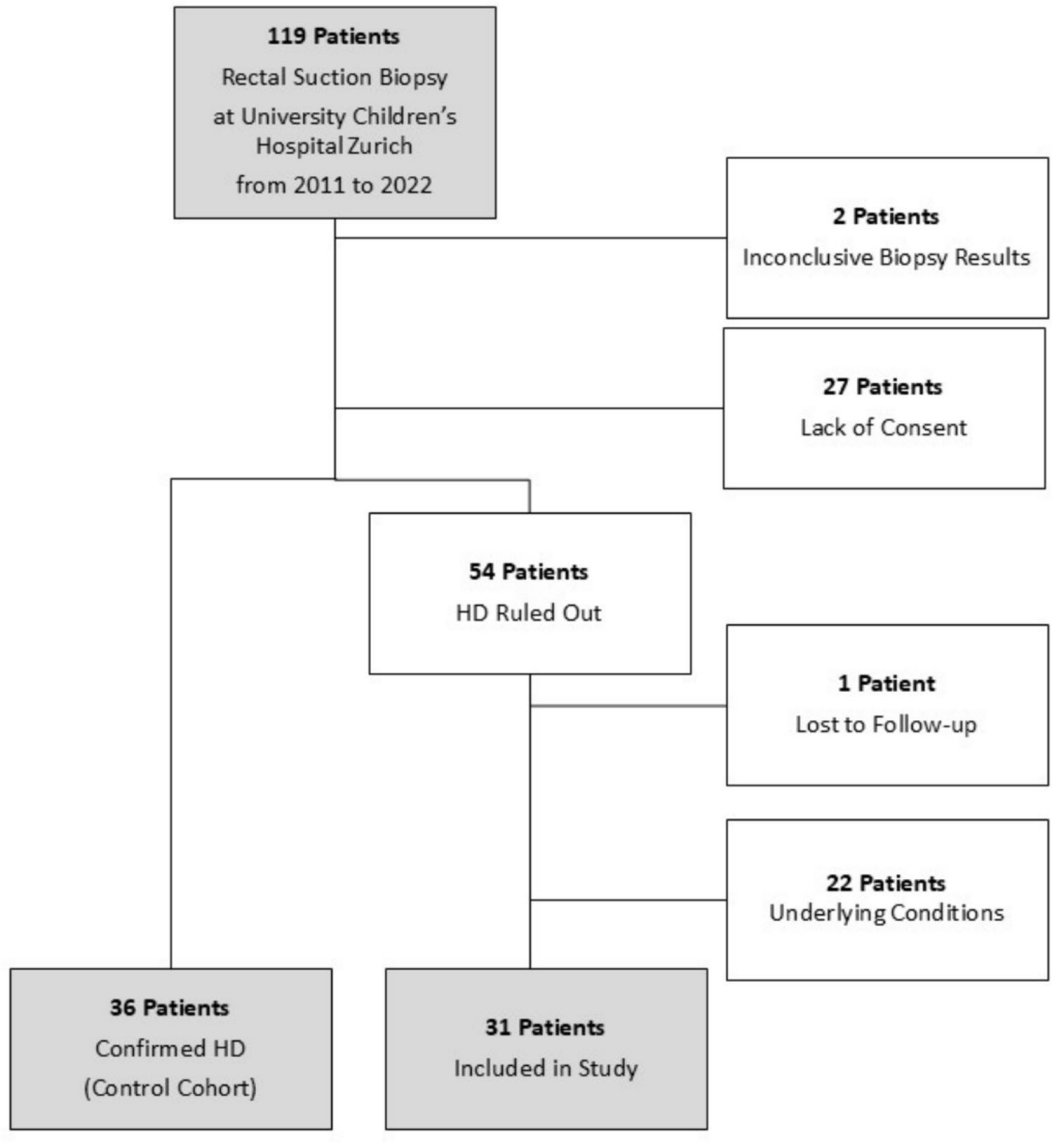
Patient selection chart

A total of 119 patients underwent rectal suction biopsies at our institution from January 2011 to May 2022. We received consent from 92 patients’ families to study their data. Thirty-six patients were diagnosed with HD, 2 patients had inconclusive biopsy results, and in 54 patients, HD could be ruled out. Twenty two of these were excluded due to other conditions, mainly gastrointestinal malformations, possibly leading to the clinical presentation (Table 1). Two patients with inconclusive biopsy results were followed up closely and were, in mutual agreement with the parents, not re-biopsied to obtain conclusive results due to an unremarkable clinical course. As HD was, by definition, not histologically excluded in these patients, they were not included in this study. One patient was lost to follow-up. Thus, 31 patients were included for further analysis with the HD patients serving as a comparison cohort (Fig. 1).

**Table 1:** Underlying conditions possibly explaining the clinical presentation and leading to exclusion of patients from the study

Condition	*n*
Intestinal perforation	4
Trisomy 21	3
Milk curd syndrome	2
Colonic atresia	2
Intestinal malrotation or midgut volvulus	3
Anorectal malformation	2
Ileum atresia	2
Meconium peritonitis	2
Gastroschisis	1
Organo-axial gastric volvulus	1

The 31 patients with negative HD biopsies included in the study had a nearly equal gender distribution, whereas the patients with HD-positive biopsies were 78% male. Most patients were term-born and of average birth weight. The median age at first biopsy in HD-negative patients was 9 weeks and the median weight at first biopsy was 5200 g, whereas the HD patients were on average younger and weighed less at the time of the first biopsy. Mean follow-up time was 16.4 months for patients with excluded HD. Table 2 shows more detailed patient characteristics.

**Table 2:** Patient characteristics

Patient characteristics	HD excluded (*n* = 31)	HD confirmed (*n* = 36)	*p *value
Age at first presentation (median, IQR) in weeks	6 (3, 11)	2 (1, 5)	< 0.001
Age at first biopsy (median, IQR) in weeks	9 (4, 17)	2 (1, 5)	< 0.001
Time of birth (*n*, %)			1
- Preterm (< 37 weeks of pregnancy)	3 (9.7%)	3 (8.3%)	
- Term (37–42 weeks of pregnancy)	28 (90.3%)	33 (91.7%)	
Sex (male, *n*, %)	16 (51.6%)	28 (77.8%)	0.038
Weight at birth (median, IQR) in gram^a^	3470 (3020, 3800)	3218 (2810, 3588)	0.247
Weight at first biopsy (median, IQR) in gram^b^	5200 (3840, 7150)	3375 (3062, 4210)	< 0.001
Symptoms at first presentation			
- Distended abdomen	25 (80.6%)	35 (97.2%)	0.043
- Bilious emesis	3 (9.7%)	20 (55.6%)	< 0.001
- Non-bilious emesis	14 (45.2%)	24 (66.7%)	0.089
- Explosive stool passage	9 (29%)	7 (19.4%)	0.401
- Delayed meconium passage	6 (19.4%)	22 (61.1%)	0.001

*IQR* interquartile range

^a^in 6 patients with excluded HD and 2 patients with confirmed HD, the weight at birth could not be verified

^b^in 2 patients with excluded HD and in 1 patient with confirmed HD, the weight at first biopsy could not be verified

### Clinical presentation

**Fig. 2:**
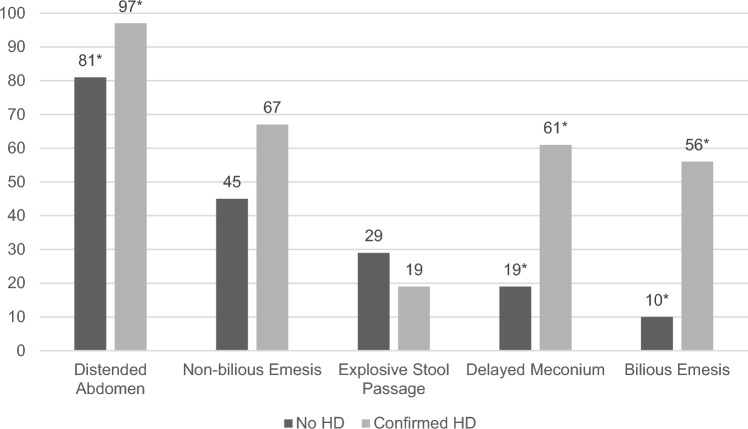
Symptoms at first presentation for patients with excluded HD and confirmed HD as comparison group (in %), *statistically significant differences (*p* < 0.05, Fisher’s exact test)

The most common symptoms at initial presentation for HD-negative patients were abdominal distension (81%, *n* = 25), followed by non-bilious emesis (45%, *n* = 14), explosive passage of stool (29%, *n* = 9), delayed passage of meconium later than 48 h after birth (19%, *n* = 6), and bilious emesis (10%, *n* = 3). In comparison, patients with confirmed HD initially presented with abdominal distension in 97% (*n* = 35), non-bilious emesis in 67% (*n* = 24), explosive passage of stool in 19% (*n* = 7), delayed meconium in 61% (*n* = 22), and bilious emesis in 56% (*n* = 20) (Fig. 2).

### Treatment

**Fig. 3:**
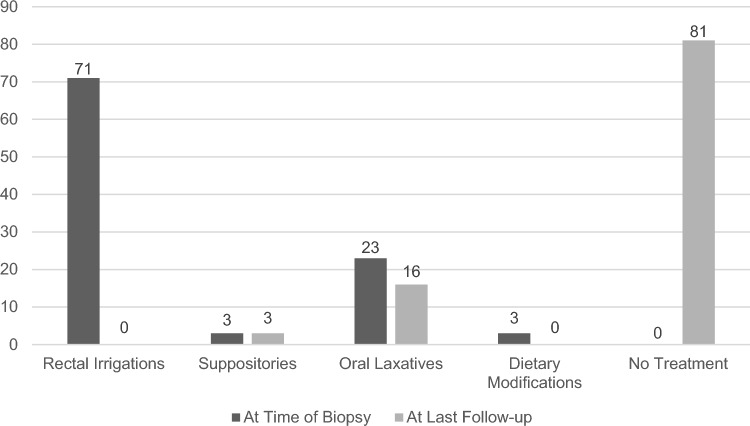
Treatment at first biopsy and at last follow-up (in %)

The following treatments were initiated prior to or immediately following biopsies for HD-negative patients: rectal irrigations in 71% (*n* = 22), suppositories in 3% (*n* = 1), oral laxatives (e.g., polyethylene glycol) in 23% (*n* = 7), and dietary modifications, i.e. hydrolyzed infant formula, in 3% (*n* = 1). Four patients (13%) received more than one treatment concurrently: Two patients (6%) were treated with suppositories and oral laxatives. One patient (3%) was administered rectal irrigations, along with oral laxative treatment. One patient (3%) regularly had rectal irrigations and suppositories. For clarity, only the more invasive treatment for each patient is shown in Fig. 3. At the last follow-up in our clinic, rectal irrigations were discontinued in all patients. One patient (3%) still regularly required suppositories, whereas 5 patients (16%) continued regularly taking oral laxatives (Fig. 3). The patient who received suppositories was also treated with hydrolyzed formula.

In two patients, we advised the referring pediatrician to promptly discontinue the rectal irrigations at the last follow-up in our outpatient clinic. No further information was received from their part, and we thus concluded that the treatments could be discontinued as planned and did not count them as still present at last follow-up.

Patients who still regularly required oral laxatives or suppositories at last follow-up show a tendency to be older at the time of first presentation and first biopsies than those who did not require any treatment at last follow-up. This is, however, not statistically significant (*p* value > 0.05). The other characteristics that were examined did not demonstrate any significant differences either (Table 3).

**Table 3:** Characteristics of HD-negative patients requiring laxatives at last follow-up

	Laxative use or suppositories at last follow-up (*n* = 6)	No treatment at last follow-up (*n* = 25)	*p* value
Time of birth (*n*, %)			1
- Preterm (< 37 weeks of pregnancy)	0 (0%)	3 (12%)	
- Term (37–42 weeks of pregnancy)	6 (100%)	22 (88%)	
Sex (male, *n*, %)	1 (17%)	15 (60%)	0.083
Age at first presentation in weeks (median, IQR)	10.5 (5.5, 21.5)	6 (3, 10)	0.191
Age at first biopsy in weeks (median, IQR)	19.5 (6, 36)	8 (4, 15)	0.238
Symptoms at first presentation			
- Distended abdomen	6 (100%)	19 (76%)	0.309
- Bilious emesis	0 (0%)	3 (12%)	1
- Non-bilious emesis	2 (33%)	12 (48%)	0.664
- Explosive stool passage	0 (0%)	9 (36%)	0.145
- Delayed meconium passage	0 (0%)	6 (24%)	0.309

### Alternative diagnoses and re-referrals

In addition to performing RSB, 13 patients (42%) in whom HD was ruled out were referred to a pediatric gastroenterologist during or after their treatment by the pediatric surgery department. Eight of these patients were referred to the gastroenterological department by the treating surgeon due to persistent symptoms after HD had been excluded. In three patients, an alternative diagnosis was found which may explain the clinical presentation: Cow’s milk protein intolerance was diagnosed in two patients, and celiac disease was suspected in one patient with positive family history and isolated elevation of anti-gliadin IgA and IgG. In the case of this patient, the diagnosis could not be confirmed, because a gluten-free diet had already been started. At last follow-up, a gluten challenge was initiated and has so far been well tolerated. No cases of hypothyroidism, cystic fibrosis, or inflammatory bowel disease were identified. Therefore, in most cases, the defecation difficulties were considered functional.

Four patients were referred again to the gastroenterology department almost 2–6 years after the end of treatment in our pediatric surgery department by the patient’s pediatrician due to new or recurring gastroenterological symptoms. All of them presented also with defecation difficulties which continued to be considered functional.

## Discussion

We report on a group of infants who underwent rectal suction biopsies due to symptoms suggestive of HD, but in whom HD was excluded. Our results suggest that the majority of these patients will experience a resolution of symptoms within the first year of life. However, there is a subset of patients who will continue to have gastrointestinal symptoms.

Given the high prevalence of gastrointestinal symptoms in otherwise healthy infants [1, 2], a careful selection of patients requiring further investigation for potentially underlying disorders such as HD is crucial. In HD patients, male sex, the presence of an underlying syndrome, a younger age at the time of RSB, delayed meconium passage exceeding 48 h, distended abdomen, bilious emesis, and failure to thrive are reported at remarkably higher frequencies [8, 18]. If there is suspicion of HD due to the patient’s history and findings on clinical examination, HD should be ruled out. The symptoms at initial presentation overlap to a considerable extent in both of our patient groups. However, the patients in whom HD was confirmed had a significantly higher frequency of distended abdomen, bilious emesis, and delayed meconium passage. Nevertheless, it seems impossible to distinguish patients with HD based on the clinical presentation alone. We therefore believe that a low threshold for histological exclusion of HD in symptomatic infants is appropriate and should be maintained.

As far as we know, there is only one other study that specifically reports on the outcome of infants in whom HD was histologically ruled out on RSB [19]. Harlev et al. describe 25 symptomatic infants with negative HD biopsies in their study, whose outcomes are comparable to those in our cohort [19]. They also noted an improvement in symptoms and a reduction in the need for laxatives over time in these patients, with prolonged laxative use in 36% of patients during the first year of life and 20% at the last follow-up (median follow-up 4.25 years). Their patients showed increased rates of constipation throughout early childhood when compared to the healthy control neonates. Additionally, they were more likely to be hospitalized or be diagnosed with a chronic gastrointestinal disease. Unlike in our cohort, they did, however, not exclude patients with underlying conditions that can lead to intestinal obstruction and constipation, such as cystic fibrosis, Noonan Syndrome, or intestinal malformations. The authors conclude that early onset constipation is associated with long-term gastrointestinal-related disorders. We cannot confirm these results to this extent. This may be because we excluded patients with underlying gastrointestinal diseases from our study. Most of our patients were free of symptoms at the final follow-up and did not require further treatment. Only four patients (13%) were referred to our gastroenterology clinic for new or recurrent gastrointestinal symptoms, and six (19%) were still taking laxatives or suppositories regularly at the final follow-up.

A number of other potential underlying diagnoses may explain defecation problems other than HD, including celiac disease, cystic fibrosis, dietary protein allergy, hypothyroidism, and anal achalasia [3]. We therefore recommend that patients who continue to have symptoms or require long-term laxative treatment should be monitored closely and be considered for referral to a pediatric gastroenterologist. As a result of this referral, in our cohort, another underlying disease leading to defecation issues was identified in three patients. Despite the long list of differential diagnoses for defecation problems in infants, the most likely diagnosis in patients with histologically excluded HD remains functional constipation [8, 9]. The pooled prevalence of functional constipation in children is estimated to be 9.5% [20]. A recent Swedish study has reported that functional constipation was observed in up to 14.3% of healthy infants during the first year of life [1]. The outcome seems to be favorable: In a follow-up study by van den Berg et al., 69% of infants with functional constipation had recovered after 6 months with no further use of laxatives, and 8% of infants showed success with the use of laxatives [21]. These numbers are comparable to those observed in our patients with negative HD biopsies. Interestingly, van den Berg’s study showed that infants in whom treatment was begun shortly after onset of symptoms had an earlier onset of success [21]. This observation may also be applicable to our cohort, as the patients who required laxatives on an ongoing basis tended to be older infants who potentially had a longer duration of symptoms before presentation. However, our study was not designed to answer this question.

Another reason why especially younger infants in our cohort showed a favorable outcome may be due to immature bowel in the first weeks of life. Immature ganglia on rectal suction biopsies have been found to be a cause for persistent symptoms of bowel obstruction in infants. These patients can usually be successfully managed conservatively until the ganglion cells mature [22]. Another review by Feichter et al. also mentions the theory of an immature bowel as the cause of constipation in children during the first year of life [23]. This theory is supported by our clinical observation in symptomatic premature infants, who in most cases, after a period of necessary rectal enemas, begin to have regular and spontaneous bowel movements over time. This is why, we rarely perform RSB in premature infants in our institution. This spontaneous improvement could be attributed to the maturation of ganglion cells as the baby approaches term, since only in a minority of preterm infants, HD is confirmed on RSB to explain the obstructive gastrointestinal symptoms [24]. In our study, we did not examine the biopsy samples specifically for immature ganglion cells. The successful clinical outcomes over time, especially in younger infants, may suggest that symptom improvement may also be related to increasing age. This could be a further indication that these young infants with potentially immature bowels represent a distinctly different patient group than older infants who develop symptoms only later in their first year of life. Such symptoms are typically associated with functional constipation and are likely to increase the risk of prolonged treatment or recurrent constipation symptoms later on in life [25].

Furthermore, histopathological diagnoses, such as intestinal neuronal dysplasia B (IND B) or hypoganglionosis, are the subject of significant controversy with regard to their role in causing symptoms of infant defecation difficulties. [10, 22]. It is debated whether these clinical and histopathological abnormalities are normal stages of bowel development or a secondary finding of long-standing constipation [10, 22]. Koletzko et al., in their review of pathological samples, showed that the rectal biopsies documented as “abnormal” were much more common in infants than in older children, leading them to hypothesize that these histopathological features may be an age-related phenomenon rather than a pathological finding [26]. However, the importance of demonstrating these histopathological features remains unclear as most patients will spontaneously have a favorable outcome, as highlighted by this study. Further studies are needed to evaluate this concept.

Due to its retrospective design, there are several limitations to this study: For one, we relied on comprehensive patient chart documentation and had to assume not mentioned symptoms as not present, which may cause bias. Follow-up consultations were not standardized. As soon as an underlying condition was reasonably ruled out or symptoms improved patients were usually referred to their pediatrician. This led to a short follow-up after biopsy in some patients as data from the general practitioners were not available. Further, we had a large number of patients who had to be excluded due to lacking consent leading to a smaller cohort.

## Conclusion

Parents may be reassured that defecation difficulties in early infancy will resolve in most patients with histologically excluded HD with no further need for treatment. However, some patients will continue to have gastrointestinal symptoms later in life. Therefore, we recommend ongoing follow-up of patients with persistent symptoms and long-term need for laxative treatment and, if necessary, referral to a pediatric gastroenterologist.

## Data Availability

Data supporting the findings of this study are not openly available due to reasons of sensitivity and are available from the corresponding author upon reasonable request.

## Abbreviations

HD: Hirschsprung disease
RSB: Rectal suction biopsy
